# A Comparative Evaluation of 2 Videolaryngoscopes as an Intubation Aid in a Simulated Difficult Airway: A Prospective Randomised Study

**DOI:** 10.5152/TJAR.2022.21285

**Published:** 2022-10-01

**Authors:** Ajay Kumar, Anupma Taluja, Bhavna Saxena, Puneet Dwivedi

**Affiliations:** 1 Department of Anaesthesia and Critical Care, Deen Dayal Upadhyay Hospital, New Delhi, India

**Keywords:** Airway management, intratracheal, intubation, laryngoscopes

## Abstract

**Objective:**

Several videolaryngoscopes have been developed for using in difficult airway. We conducted this study to evaluate the performance of McGrath^®^ MAC and King Vision^®^ videolaryngoscopes in a simulated difficult airway.

**Methods:**

This prospective, randomised, comparative study was conducted in 140 surgical patients. Anaesthesia was administered as per standard protocol. A cervical collar was applied to simulate a difficult airway. Patients were randomised into 2 groups. In group M (n = 70), laryngoscopy was performed first with King Vision^®^ videolaryngoscope and second time with McGrath^®^ MAC videolaryngoscope and trachea was intubated using the second device, while in group K (n = 70), laryngoscopy was performed ﬁrst with McGrath^®^ MAC videolaryngoscope and second time with King Vision^®^ videolaryngoscope and trachea was intubated using the second device. The laryngeal view, time taken for optimal laryngeal view, number of intubation attempts, ease of intubation, first attempt intubation success, time to tracheal intubation, haemodynamic parameters, and complications such as airway trauma, if any, were noted.

**Results:**

Tracheal intubation was faster with McGrath^®^ MAC (34.89 ± 3.7 seconds) compared to King Vision^®^ videolaryngoscope (43.43 ± 4.3 seconds, *P*  < .001) with comparable first attempt intubation success by 100% vs 97.1%, *P*  = .496, respectively. The laryngeal view obtained with both the devices was comparable but the mean time taken for optimal laryngeal view was significantly longer with King Vision^®^ videolaryngoscope, both in group M (*P*  < .001) and group K (*P*  < .001). Ease of intubation and complications were comparable in the 2 groups.

**Conclusion:**

McGrath^®^ MAC videolaryngoscope in comparison to King Vision^®^ videolaryngoscope resulted in a shorter time taken for optimal laryngeal view and time to tracheal intubation with comparable first attempt intubation success.

**Main Points**

- Restriction of cervical spine motion is desirable while performing airway procedures in a patient with known or suspected cervical spine injury.

- Cervical collar application makes tracheal intubation using conventional laryngoscopy difficult.
- Videolaryngoscopes (VL) improve glottic view and increase intubation success.
- In a simulated difficult airway situation created by cervical collar application, McGrath^®^ MAC and King Vision^®^ (channelled) VLs provided a comparable laryngeal view and first attempt intubation success.
- McGrath^®^ MAC VL resulted in a shorter time to laryngeal view and tracheal intubation compared to King Vision^®^ (channelled) VL.

## Introduction

Airway management has always been a prime concern to the anaesthesiologist. Both anticipated and unanticipated difficulties may be experienced during laryngoscopy and intubation. Devices such as fibreoptic bronchoscopes, lighted stylets, videolaryngoscopes (VL), and various supraglottic devices are useful in managing a difficult airway. Videolaryngoscopes have combined features of both classic laryngoscopes and fibreoptic bronchoscopes, they improve glottic view, and thus increase intubation success rates and decrease anaesthesia-related morbidity and mortality.^1,2^ Currently, several types of VLs such as the King Vision^®^, McGrath^®^ MAC, Airtraq™, GlideScope™, and C-MAC™ are being marketed. The performance of these devices has been evaluated in manikin and clinical studies in varied scenarios.^1,3-7^

In patients with immobilised cervical spine, previous authors found that King Vision^®^ (channelled) VL resulted in better glottis visualisation, easier tracheal intubation, and higher first attempt success rate as compared to Macintosh and McCoy laryngoscopes.^8^ In a simulated difficult airway using a semi‐rigid collar, the McGrath^®^ Series 5 VL significantly improved the laryngeal view and was also superior in terms of intubation success, with a significantly lower intubation failure rate when compared with the Macintosh laryngoscope.^9^ The McGrath MAC VL as compared to Optiscope video stylet showed a higher first-attempt intubation success and a shorter intubation time in patients with immobilised cervical spine during tracheal intubation.^10^ There is paucity of literature regarding studies that evaluate the performance of McGrath^®^ MAC and King Vision^®^ VL (channelled blade) in a difficult airway. We thus conducted this study in a simulated difficult airway situation, created by application of a cervical collar, in adult patients to determine which of the two VLs namely McGrath^®^ MAC VL and King Vision^®^ VL is a better intubation aid in terms of time taken for tracheal intubation and first attempt intubation success (primary outcome) and hypothesised that McGrath^®^ MAC VL is a better intubation aid as compared to King Vision^®^ VL.

## Methods

The study was approved by the Ethics Committee of DeenDayal Upadhyay hospital, Government of NCT of Delhi and was registered with Clinical Trials Registry-India (CTRI/2017/08/009380). A written informed consent was obtained from all patients following a detailed explanation of the study procedure.

**Figure 1.**
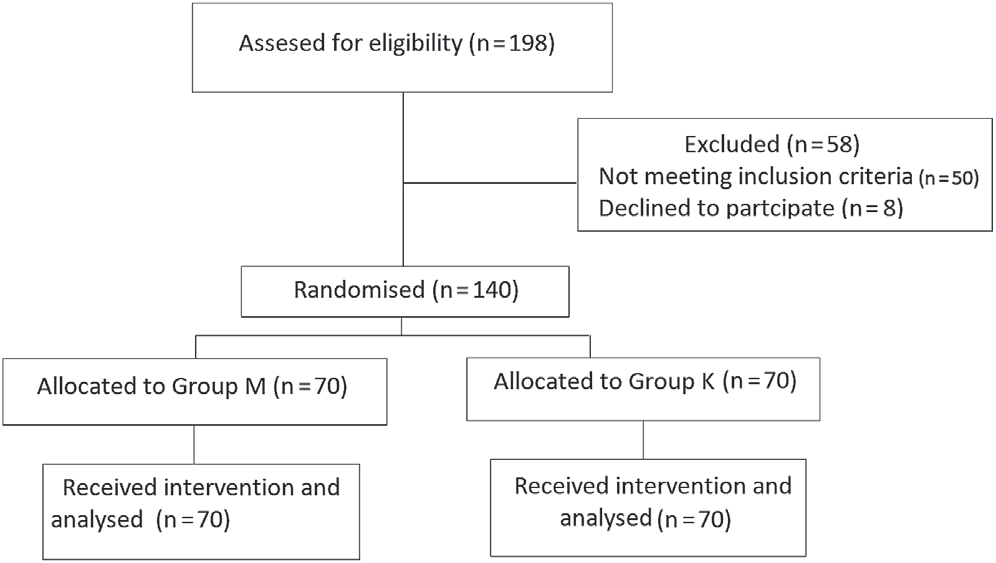
Consolidated Standards of Reporting Trials (CONSORT) diagram.

A total of 198 patients were assessed for eligibility. Of these, 50 patients did not meet the inclusion criteria and 8 patients declined to participate. Finally, 140 patients were randomised into the 2 study groups, with 70 patients in each group (Figure 1).

This prospective, randomised, comparative study was conducted in 140 adults, aged 18-60 years, of either gender, with American Society of Anaesthesiologists physical status grade I or II, undergoing elective surgery, under general anaesthesia requiring orotracheal intubation. Patients with known or anticipated difficult airway, modified Mallampati class (MMC) 3 or 4, thyromental distance (TMD) < 6 cm, inter-incisor distance (IID) < 3.5 cm, restricted neck mobility, oral or laryngeal pathology, cervical spine injury or disease, loose or absent front dentition, cardiovascular or respiratory disease with risk factors for gastric aspiration such as obesity, pregnancy, and diabetes were excluded from the study.

All patients fasted overnight and received tablet alprazolam of 0.25 mg night before and 2 h prior to surgery. Using a computer-generated random number table, patients were allocated randomly into 2 groups, group K (n = 70) and group M (n = 70) in which King Vision^®^ VL (size 3 channelled blade) and McGrath^®^ MAC VL (size 3 blade), respectively, were used for tracheal intubation, in a simulated difficult airway situation created by application of a cervical collar. Concealment of allocation was done using sealed, opaque envelopes.

In the operation theatre, standard monitors (electrocardiograph, non-invasive blood pressure and pulse oximeter) were applied and baseline readings were noted. Anaesthesia was induced with fentanyl 2 μg kg^−1^ and propofol 2 mg kg^−1^. After confirming adequate bag-mask ventilation, vecuronium bromide of 0.1 mg kg^−1^ was administered for neuromuscular blockade. Once muscle relaxation was achieved, the IID at maximal mouth opening was measured using a vernier calliper. To simulate a difficult airway, an adjustable one-piece rigid cervical collar (Ambu^®^ Perfit ACE) was applied. The IID was again measured and noted (aiming 2.5 cm).

The anaesthesiologist who inserted the study device had received prior training in the use of both the VLs on a manikin and subsequently used them clinically at least 20 times or more, until felt competent in using each device. In group M, laryngoscopy was ﬁrst performed with King Vision^®^ VL, first device (D1). Laryngeal view was assessed using modified Cormack Lehane (CL) grade and percentage of glottic opening (POGO) score. The time taken to obtain optimal laryngeal view (TLV) was noted. Lungs were ventilated with 100% oxygen, and thereafter, second device (D2), McGrath^®^ MAC VL, was used to perform laryngoscopy. Laryngeal view and TLV were noted. Trachea was intubated using this second device and time taken for tracheal intubation (TTI) was noted. While in group K, the process was reversed, and laryngoscopy was ﬁrst performed with McGrath^®^ MAC VL, first device (D1). Laryngeal view and TLV were noted. Lungs were ventilated with 100% oxygen, and thereafter, second device (D2), King Vision^®^ VL, was used to perform laryngoscopy. After noting the laryngeal view and TLV, trachea was intubated using this second device and TTI was noted. Tracheal intubation was done with tracheal tube size 7 for females and 8 for males. In group M, a stylet lubricated with water-based lubricant was inserted into the tracheal tube and the tube was bent, keeping it opposed to the blade, to facilitate tracheal intubation with McGrath^®^ MAC VL. While in group K, the tracheal tube was preloaded in the blade channel and inserted through the leading channel to facilitate tracheal intubation with King Vision^®^ VL.

Correct placement of tracheal tube was confirmed by capnography and chest auscultation for bilateral breath sounds. Anaesthesia was maintained using vecuronium bromide, nitrous oxide (66%), and isoflurane in oxygen. At the end of surgery, residual neuromuscular blockade was reversed with neostigmine and glycopyrrolate.

Time taken to obtain optimal laryngeal view was defined as time from introduction of VL between the incisors to obtaining optimal laryngeal view, (centralising the vocal cords in view) and TTI was defined as the time from introduction of VL between incisors to appearance of the capnograph tracing. If tracheal intubation failed in the first attempt, a second intubation attempt was taken. The duration of second intubation attempt was added to the TTI. An intubation attempt was defined as each time the tracheal tube was newly advanced towards the glottic opening. Only two attempts were allowed for tracheal intubation. The study was terminated, the cervical collar was removed, and alternative airway management plan was instituted after two failed intubation attempts (device failure) or upon occurrence of airway injury, bronchospasm, technical failure, or SpO_2_ < 90%.

Patients were monitored throughout the surgical procedure. Readings of heart rate, systolic, diastolic, and mean blood pressure and peripheral oxygen saturation were noted at the following time points: baseline, after induction, first study device insertion, second study device insertion, intubation, and 1, 3- and 5-min post-intubation. Laryngeal view was assessed using modified CL grade (grade1: most cords visible; 2a: posterior cords visible; 2b: only arytenoids visible; 3a: only epiglottis visible and liftable, 3b: epiglottis adherent to pharynx; and 4: no laryngeal structures seen. Grades 1 and 2a were graded as easy, 2b and 3a as restricted, and grade 3b and 4 as difficult view at laryngoscopy^11^ and the POGO score was noted (100% for full view of glottis from the anterior commissure to the interarytenoid notch and score 0 for inability to visualize even the interarytenoid notch).^12^

Ease of intubation was graded on a scale from 0 (extremely easy) to 10 (extremely difﬁcult).

Time taken for tracheal intubation (TTI) and first attempt intubation success (primary outcome), laryngeal view, TLV, number of intubation attempts, ease of intubation, haemodynamic parameters, and complications such as dental damage, blood on the laryngoscope blade, airway trauma, if any, were noted (secondary outcomes).

### Statistical Analysis

Using one-tailed alpha value (0.05) and beta value (0.2), 70 patients per group were considered sufficient, based on a previous study^1^ to detect a significant difference of 11% between 2 groups (87% in King Vision^®^ group vs 98% in McGrath^®^ MAC group) with respect to first attempt success in tracheal intubation. Descriptive statistics were analysed with the Statistical Package for the Social Sciences version 17.0 software (SPSS Inc.; Chicago, IL, USA). Continuous variables were presented as mean ± SD or median (IQR) for non-normally distributed data. Categorical variables were expressed as frequencies and percentages. The comparison of normally distributed continuous variables between the groups was performed using Student’s *t* test. Nominal categorical data between the groups were compared using chi-square test or Fisher’s exact test as appropriate. Non-normal distribution of continuous variables was compared using Mann–Whitney *U* test. *P  <  *0.05was considered statistically significant.

## Results

The patient and airway characteristics in the 2 study groups are as shown in Table 1. The mean POGO score and modified CL grade with each of the 2 devices were comparable in both groups M and K (Table 2). First attempt intubation success was comparable in the 2 groups, *P*  =  .496, (Table 3). There was no device failure in any group. The mean TLV was significantly longer with device King Vision^®^ in both group M (*P*  < .001) and group K (*P*  < .001) (Table 2). The mean TTI was significantly longer with device King Vision^®^ VL (43.43 ± 4.3 seconds) as compared to McGrath^®^ MAC VL (34.89 ± 3.7 seconds) (Table 3). The ease of intubation was comparable in the 2 groups (Table 3). There were only minor complications, namely, lip trauma observed in 1 (1.4%) and 2 (2.9%) patients in group M and K, respectively.

**Figure 2.**
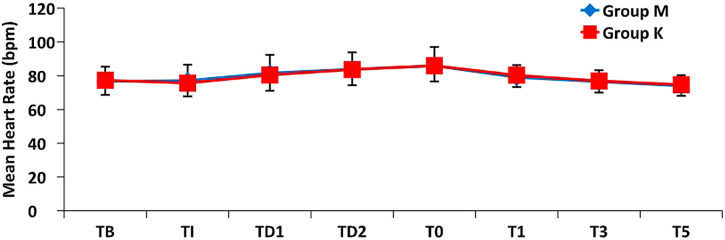
Heart rate changes at various timepoints in the 2 groups. X axis is time points: TB, baseline; TI, induction; TD1, first study device insertion; TD2, second study device insertion; T0, intubation; T1, T3, and T5 are 1, 3, and 5 minutes post-intubation.

**Figure 3.**
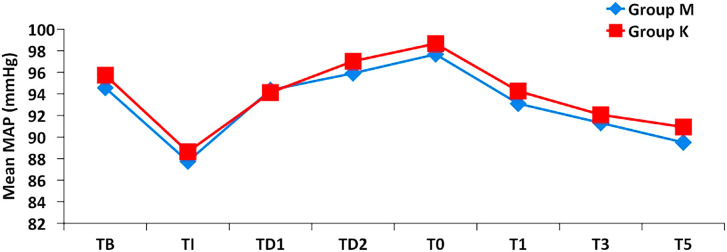
Mean arterial pressure (MAP) changes at various timepoints in the 2 groups. X axis is time points: TB, baseline; TI, induction; TD1, first study device insertion; TD2, second study device insertion; T0, intubation; T1, T3, and T5 are 1, 3, and 5 minutes post-intubation.

All patients in both the groups remained haemodynamically stable with minimal haemodynamic changes throughout the study. The heart rate and mean arterial pressure (mean values) were comparable in the 2 groups at all time points (Figure 2 and 3). No patient had oxygen desaturation throughout the procedure.

## Discussion

Restriction of cervical spine motion while performing airway procedures, by manual in-line stabilisation or cervical collar application, is desirable in patients with known or suspected cervical spine injuries. Cervical collar application not only restricts cervical spine motion but also limits mouth opening, making tracheal intubation using conventional laryngoscopy even more difficult, with a reported success rate of only 40% with Macintosh laryngoscope.^13^ In such a situation, use of VLs may be beneficial as they allow a wide viewing angle and make alignment of oral, pharyngeal, and tracheal axes unnecessary.^14^ In a meta-analysis by Suppan et al^15^ patients with cervical spine immobilisation had a lower risk of intubation failure with VLs as compared to Macintosh laryngoscope.

We simulated a difficult airway using a cervical collar to restrict head and neck movement and reduce mouth opening to 2.5 cm and evaluated the performance of McGrath^®^ MAC and King Vision^®^ VL. We found that tracheal intubation was significantly faster with McGrath^®^ MAC VL (34.89 ± 3.7 seconds) compared to King Vision^®^ VL (43.43 ± 4.3 seconds ) with comparable first attempt intubation success of 100% vs 97.1%, respectively. We were unable to intubate the trachea in 2 patients in the first attempt with King Vision^®^ VL, due to drifting of the tracheal tube towards the right arytenoid upon advancing it towards the glottis. In both these cases, VL blade required realignment, to allow the passage of tracheal tube through the glottis. The possible reason for impingement occurring at the right aryepiglottic fold could be due to central insertion of device and introduction of the tracheal tube from the right of the device.^16^

Previous investigators found a significantly shorter time to tracheal intubation and a higher first attempt success rate with McGrath MAC VL compared to King Vision VL.^17^ The median time for successful intubation was shorter with McGrath MAC VL compared to the King Vision VL (17 vs 38 seconds).^17^ In our study, the TTI with both the devices was longer than that observed by Alvis et al.^17^ This could be because of the difference in the method of calculating TTI, besides our study population had a difficult airway situation created by applying a cervical collar unlike Alvis et al^17^ whose study population had a predicted easy intubation. Shah et al^16^ observed that the TTI using King Vision VL (channelled blade) was found to be only 15.24 ± 10.6 seconds. A shorter TTI could be because cervical spine immobilisation was not done in their study population unlike ours and due to the difference observed in the method of calculating TTI.

In a simulated difﬁcult airway, using manual in-line stabilisation, Taylor et al^18^ found mean TTI to be 35.8 ± 20.4 seconds with McGrath^®^ Series 5 VL, similar to our study. Other authors found the TTI with McGrath^®^ VL was 30.02 ± 9.87 seconds with 100% first attempt intubation success in patients with simulated cervical spine injury with application of manual in-line stabilisation.^19^ In patients with a simulated difﬁcult airway with a cervical collar, the median intubation time with McGrath^TM^ MAC compared to King Vision^TM^ (channelled blade) was 53 vs 59 seconds; *P*  < .01.^1^

In our study, the factors that contributed to shorter TTI with McGrath™ VL included the ease with which lighter unchannelled blade of McGrath^®^ MAC VL could be inserted into the mouth and manoeuvred as compared to King Vision^®^ channelled blade that was bulky and more difficult to insert into the mouth. In some cases, we experienced difficulty in negotiating the tracheal tube through the channel in King vision^®^ blade into the glottis as the tip of the tube drifted towards the right arytenoid. This required manipulation of the device. Previous investigators have found that manipulation of the device was needed in most patients (69%) where impingement occurred in the case of a channelled blade King Vision™ videolaryngoscope.^16^

The similarity in McGrath^®^ Mac blade design to that of classic Macintosh laryngoscope blade also increased the intubator’s comfort and perhaps contributed to a shorter TTI with it.

Laryngeal view assessed using modified CL grade and POGO score with McGrath^®^ MAC and King Vision^®^ VL were comparable, in both the groups. We found that mean POGO score with King Vision^®^ VL was 98.54 ± 6.94 and 99.37 ± 4.22 in groups M and K, respectively, while mean POGO score with McGrath™ Mac VL was 100.00 ± 0.00 in both groups M and K. Previous investigators in a simulated difficult airway with cervical collar application found median POGO score of 90 with both King Vision^TM^ and McGrath^TM^ VL and noted CL grading I/IIa/IIb/III/IV in 64/45/9/1/0 patients with McGrath^TM^ and 63/41/7/1/4 patients with King Vision^TM^ VL out of 120 patients studied in each group.^1^ In our study, all patients in both the groups had modified CL grading of I or IIa and were classified as having easy laryngoscopy view.

Time taken to obtain optimal laryngeal view was significantly less with McGrath^®^ MAC VL as compared to King Vision^®^ VL in both group M (9.86 ± 2.08 vs 11.93 ± 2.82) and group K (10.19 ± 1.95 vs 13.84 ± 2.48). Other authors have also reported lesser median time for laryngoscopy with McGrath^TM^ MAC as compared to King Vision^TM^ VL 18 vs 26 seconds, respectively.^1^ The median time required to obtain adequate laryngeal view with McGrath^®^ MAC VL has been reported as 6.3 seconds [interquartile range, 4.7-8.7 (range,2-26.3)].^20^

Ease of intubation is a subjective criterion, though we felt intubation was easier with McGrath^®^ MAC VL, the 2 groups were comparable with respect to ease of intubation. [Table t3-tjar-50-5-340]None of the patients in either group had complications other than lip trauma.

Our study had some limitations. It was not possible to blind the operator to the type of VL used. The ease of intubation is a subjective measure. It is possible that the performance of VL varies depending on the type of difficult airway, and our results may not extrapolate to other difficult airway situations. Further studies are required to elucidate the performance of these devices in other difficult airway situations as well.

## Conclusion

In adult patients, a simulated difficult airway situation created by cervical collar application McGrath^®^ MAC and King Vision^®^ (channelled) VLs had comparable first attempt intubation success with no device failure, comparable ease of intubation, and minimal complications. However, laryngoscopy and tracheal intubation were faster with McGrath^®^ MAC VL as compared to King Vision^®^ (channelled) VL.

## Figures and Tables

**Table 1.** Patient and Airway Characteristics

Parameter	Group M (n = 70)	Group K (n = 70)	*P*
Age (year)	37.01 ± 10.34	37.36 ± 9.77	.840
Male/female (n)	25/45	21/49	.472
Height (cm)	159.41 ± 7.25	157.57 ± 6.57	.117
Weight (kg)	65.34 ± 8.15	65.53 ± 7.83	.891
BMI (kg m−^2^)	25.66 ± 2.37	26.38 ± 2.66	.093
ASA grade I/II (n)	60/10	61/9	.805
MMC I/II (n)	49/21	53/17	.447
TMD (cm)	7.74 ± 0.68	7.88 ± 0.63	.212
IID (cm)	4.49 ± 0.38	4.42 ± 0.37	.246

Values are mean ± SD or as numbers

BMI, body mass index; ASA, American Society of Anesthesiologists; MMC, modified Mallampati class; TMD, thyromental distance; IID, inter-incisor distance; SD, standard deviation.

**Table 2.** Grading Laryngoscopy View and Time Taken for Obtaining Optimal View

Parameter	Group M (n = 70)			Group K (n = 70)		
D1 KV	D2 McGrath	*P*	D1 McGrath	D2 KV	*P*
Modified CL grade 1/2a/2b/3a/3b/4	68/2/0/0/0/0	70/0/0/0/0/0	.496	70/0/0/0/0/0	68/2/0/0/0/0	.496
POGO Score	98.54 ± 6.94	100.00 ± 0.00	.083	100 ± 0.00	99.37 ± 4.22	.217
TLV (seconds)	11.93 ± 2.82	9.86 ± 2.08	<.001	10.19 ± 1.95	13.84 ± 2.48	<.001

Values are mean ± SD or as numbers

CL, Cormack Lehane; POGO, percentage of glottic opening; TLV, time taken for optimal laryngeal view; KV, KingVision; SD, standard deviation.

**Table 3. t3-tjar-50-5-340:** Intubation Characteristics

Parameter	Group M (n = 70)	Group K (n = 70)	*P*
Intubation attempt (1/2)	70/0	68/2	.496
TTI (seconds)	34.89 ± 3.7	43.43 ± 4.3	<.001
Device failure	0	0	1.000
Ease of intubation score 0/1/2/3/4/5/6/7/8/9/10	70/0/0/0/0/0/0/0/0/0/0	62/0/4/2/1/1/0/0/0/0/0	.075

Values are mean ± SD or as numbers

TTI, time taken for tracheal intubation; SD, standard deviation.
